# Quality Prediction and Control in Wire Arc Additive Manufacturing via Novel Machine Learning Framework

**DOI:** 10.3390/mi13010137

**Published:** 2022-01-15

**Authors:** Xinyi Xiao, Clarke Waddell, Carter Hamilton, Hanbin Xiao

**Affiliations:** 1 Mechanical and Manufacturing Engineering Department, Miami University, Oxford, OH 45056, USA; xiaox8@miamioh.edu (X.X.); waddelc2@miamioh.edu (C.W.); 2 School of Transportation and Logistics Engineering, Wuhan University of Technology, Wuhan 430062, China

**Keywords:** wire arc additive manufacturing (WAAM), quantitative process-quality analysis, novel machine learning framework

## Abstract

Wire arc additive manufacturing (WAAM) is capable of rapidly depositing metal materials thus facilitating the fabrication of large-shape metal components. However, due to the multi-process-variability in the WAAM process, the deposited shape (bead width, height, depth of penetration) is difficult to predict and control within the desired level. Ultimately, the overall build will not achieve a near-net shape and will further hinder the part from performing its functionality without post-processing. Previous research primarily utilizes data analytical models (e.g., regression model, artificial neural network (ANN)) to forwardly predict the deposition width and height variation based on single or cross-linked process variables. However, these methods cannot effectively determine the optimal printable zone based on the desired deposition shape due to the inability to inversely deduce from these data analytical models. Additionally, the process variables are intercorrelated, and the bead width, height, and depth of penetration are highly codependent. Therefore, existing analysis cannot grant a reliable prediction model that allows the deposition (bead width, height, and penetration height) to remain within the desired level. This paper presents a novel machine learning framework for quantitatively analyzing the correlated relationship between the process parameters and deposition shape, thus providing an optimal process parameter selection to control the final deposition geometry. The proposed machine learning framework can systematically and quantitatively predict the deposition shape rather than just qualitatively as with other existing machine learning methods. The prediction model can also present the complex process-quality relations, and the determination of the deposition quality can guide the WAAM to be more prognostic and reliable. The correctness and effectiveness of the proposed quantitative process-quality analysis will be validated through experiments.

## 1. Introduction

Additive manufacturing (AM) is widely used in today’s manufacturing industry to build geometries in a layer-by-layer material deposition manner instead of subtracting materials from a monolithic volume. The advantages of such a process go beyond just the geometric complexity and the wide range of material selection. The deposition manner additionally improves the manufacturability rather than being hindered by the machining tool accessibility. One such metal AM technique is wire arc additive manufacturing (WAAM), which can produce large metal components using various materials, such as titanium, aluminum, and nickel-based alloys [1,2,3], for the automobile and aerospace industries. However, these advantages are typically realized at a lower resolution and dimensional accuracy. Specifically, for the layer-by-layer material accumulation, the inaccuracy of the deposition bead can cause error accumulation while depositing additional layers. Thus, the accuracy of the single bead shape is critical to ensure the overall deposition geometry and to prevent any process anomalies, such as collisions and over/lack-of build.

**Figure 1:**
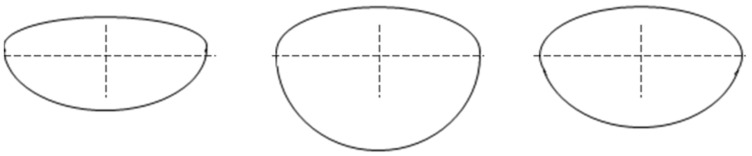
Varied deposited bead shape based on the different process parameters.

Recently, some significant research in WAAM characterized the microstructure of low carbon steel walls [4] and utilized multi-degree-of-freedom in WAAM deposition to explore freeform additive capabilities [5,6]. Additionally, other researchers have analyzed the tensile strength from different material deposition systems [7]. However, if a functional component is desired from the WAAM process, one of the critical aspects is to ensure that the deposition shape fits the desired input model. For example, in Figure 1, different processing conditions can result in different deposited bead shape and depth of penetration (DOP), ultimately affecting the final deposition quality in terms of shape, microstructure, and mechanical properties. The deposited bead geometry varied based on the following process parameters: welding current, voltage, contact-to-workpiece distance, and travel speed [8]. The WAAM process variables of current and wire feed rate are interrelated, so one cannot be independently adjusted. Thus, the prediction of the deposited bead shape from selecting the process parameters is an essential aspect of ensuring the success of the near-net-shape deposition. In addition, creating a printable zone, which determines the optimal selections and combinations of varied and interconnected process parameters, can improve the deposition quality.

In Figure 1, the deposited bead geometry varied based on the following process parameters: welding current, voltage, contact-to-workpiece distance, and travel speed [8].

Table 1 presents related research that discusses the process parameters’ effect on the ultimate build qualities [9,10,11,12,13,14,15,16,17,18,19,20]. The significant parameters that affect the as-desired build qualities are widely discussed. However, the specific quantitative relationship between these parameters and the desired qualities is not fully explored, preventing the full control of the WAAM build quality.

Existing researchers have already discussed the primary effects of welding current and welding voltage variables on the weld penetration profile [9]. However, the specific analytical relationship has not been fully discovered to create a desired weld bead geometry and properties for WAAM deposition. In general, traditional response surface modeling uses either one or the mixture model of first- and second-order models. The models are represented below:

$$Y=C_{0}+C_{1}*X_{1}+C_{2}*X_{2}+\ldots +C_{n}*X_{n}$$

$$\begin{array}{l} Y=C_{0}+C_{1}*X_{1}+C_{2}*X_{2}+C_{12}*X_{1}*X_{2}+C_{11}*X_{1}^{2}+C_{22}*X_{2}^{2}+\ldots +C_{n}*X_{n}+C_{m}*X_{m}+C_{\mathrm{mn}}*X_{n}*X_{m}\\ \;\;\;\;\;\;+C_{\mathrm{nn}}*X_{n}^{2}+C_{\mathrm{mm}}*X_{\mathrm{mm}}^{2} \end{array}$$

where $x_{n}$ is the variable that can control the response *Y*; $C_{n}$ and $C_{n,m}$ are fitting coefficients. Once the data are collected, the least square method is used to estimate the proper coefficients; however, the traditional response surface modeling method can only predict the response based on the selection of the process variables ($x_{n}$). This model cannot reversely define the optimal selection of the variables based on a desired response level.

**Figure 2:**
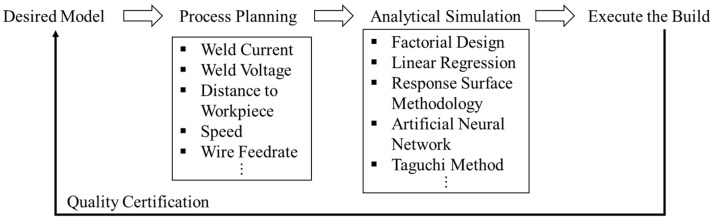
WAAM workflow and analytical approaches.

Figure 2 presents the current workflow from the selected input model to the physical building process. This workflow compiles the available statistical approaches to analyze the relationship among various WAAM processing conditions, the weld bead, and related mechanical properties. Figure 2 shows that the build quality can be determined by selecting the variables in the process planning stage that need to be examined by comparison with the desired input geometries. However, due to the limited understanding of the correlated process parameters and their effects on the deposited bead shape, it is difficult to evaluate the incorporated controlling effects of the influential process parameters in WAAM.

Given the input variants {$x_{i}$} and response y, the multi-dimensional response model can be formalized as:

$$y=f\left(x_{1},x_{2},\ldots ,x_{i}\right)$$

However, the covariant relationships among {$x_{i}$} remains anonymous. The mathematical function for representing the system response model is hard to outline. Typically, a surrogate model/machine learning method is adopted to describe the relationship between these correlated process parameters and the final response. However, the methods have the following limitations that are not suitable for developing a printable zone based on the desired deposition bead shape:

1. A surrogate model contains one response but with multiple process variables. It is difficult to reversely infer and indicate the domain of the process variable based on single/multiple response data (e.g., $x_{i}=f^{-1}\left(y_{1},y_{2},x_{1},x_{2},\ldots )\right)$ ($x_{i}$: process variables, y: process response).
2. A surrogate model primarily focuses on the possibility of the response deviation and cannot provide a numerical level of such deviation.
3. Machine learning models, such as k-nearest neighbors (KNN), naïve Bayes mainly focus on predicting the categorical response. Therefore, the quantitative analysis of the desired bead shape is not achievable.
4. Other machine learning models, such as response surface methodology (RSM), dominantly use a second-degree polynomial model to indicate the input-output relationship. Still, the reverse indication of the input from the desired output cannot be obtained.

With the abovementioned limitations of the existing methodologies, WAAM is difficult to control and predict and can exhibit low process repeatability [21]. Therefore, a quantitative process-quality model that describes the influence of individual process variables and their correlated impacts is urgently needed to better control print quality. To this end, a multi-dimensional neural network framework is developed in this paper to draw such a relationship. The quantitative process-quality relationship between various process parameters and deposition bead shape is obtained and evaluated to measure and control geometry as near-net-shape.

The proposed machine learning framework is capable of quantitatively predicting the response and reversely indicating the optimal printable zone via the deducible process-quality network. In contrast, the traditional surface response model (RMS) for the process-quality modeling cannot describe the response which integrates more than two variables. The objectives of this research are stated as follows:

1. Develop a quantitative process-quality machine learning framework of influential process parameters towards deposition quality for stainless steel on the WAAM process.
2. Classify the correlated process parameters and construct a qualitative model towards the deposition shape level from the correlated parameters.
3. Printable zone development is based on quantitative models in the network that control the deposition shape at a certain level.
4. A predictive model for controlling the deposited bead shape (width, height, and penetration depth) based on sets of input process parameters.

## 2. Methodology

**Figure 3:**
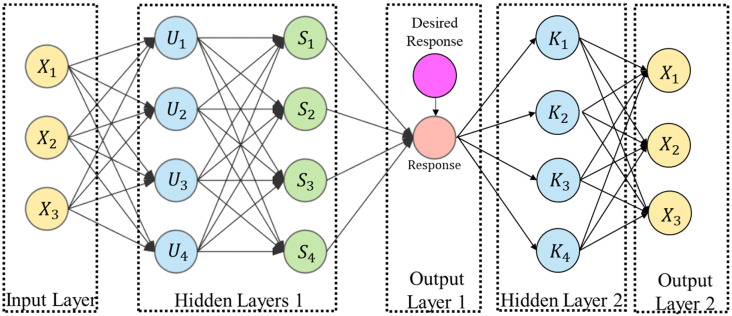
A machine learning framework for quantitatively analyzing deposition shape.

In this paper, wire feed rate is not considered in building the quantitative process-quality analysis since it is highly dependent and can be reflected by the change of weld current [22]. This paper proposes a novel machine learning framework that involves three process variables (current, voltage, and speed) to build a process-quality quantitative model to control the deposited bead shape. A multi-dimensional variability neural network model, driven by the machine learning framework (Figure 3) for recognizing the system multi-process variability, is developed as a process-quality model for predicting and controlling the bead shape in WAAM.

Figure 3 presents the proposed machine learning framework, including the forward prediction of the deposition shape by selecting process inputs, that is capable of reversely deducing optimal process inputs. The structure in the proposed neural network consists of (a) the number of hidden layers, (b) the number of neurons in each layer, (c) the activation function, and (d) the loss estimation function [23]. The activation transformation based on the input layers is vital to the neural network model; the activation functions in the hidden layers are:

$$U_{k}=\cup^{\;}P_{k}:u(\sum^{\;}X_{i})<P_{k}<v(\sum^{\;}X_{i})$$

$$S\left(u,v\right)=\sum_{m=1}^{k}\sum_{n=1}^{l}R_{m,n}\left(u,v\right)W_{k}U_{k}$$

The root means square (*RMS*) is adopted to represent the loss function that determines the difference between the predicted response value and the targeted value, and the mathematical expression is given as:

(1) $$RMS=\sqrt{\frac{\sum_{i=1}^{n}{\left(response_{i}-response_{t}\right)}^{2}}{n}}$$

where i is the index, $response_{i}$ is the predicted value in the network, and $response_{t}$ is the target value.

**Figure 4:**
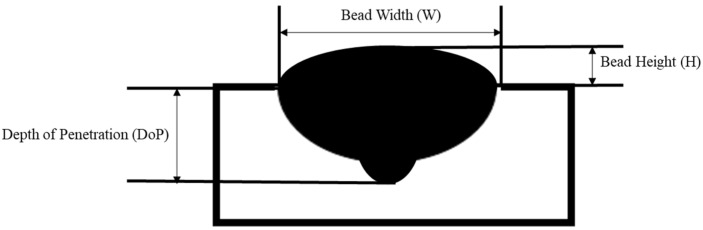
Deposited bead shape visualization.

The experimental data collected for this study was gathered with a metal inert gas (MIG) welding robot using AISI 420 stainless steel. The WAAM deposition process is performed with preheating. The bead was deposited on the 160 mm substrate. Subsequently, the cross section of the deposited bead was measured. As proposed by other research [14], the design of the experiment and the response surface methodology were used to obtain the statistical analysis to investigate the significant level of the process parameter to the deposited bead shape. However, these methods cannot reversely indicate the optimal selection of the process parameters to achieve the desired deposition shape. Therefore, a novel process-quality neural network model is proposed in this section and used as a predictive method to determine the relationship between the input (current, voltage, and travel speed) and the deposited bead shape (width, height, and DOP). In addition, a printable zone includes the optimal process parameters for achieving the desired deposition bead shape can be constructed. Such printable zone can navigate the printability and improve the metal AM repeatability. Figure 4 visualizes the deposited bead, including the parameters that are analyzed in this study.

**Figure 5:**
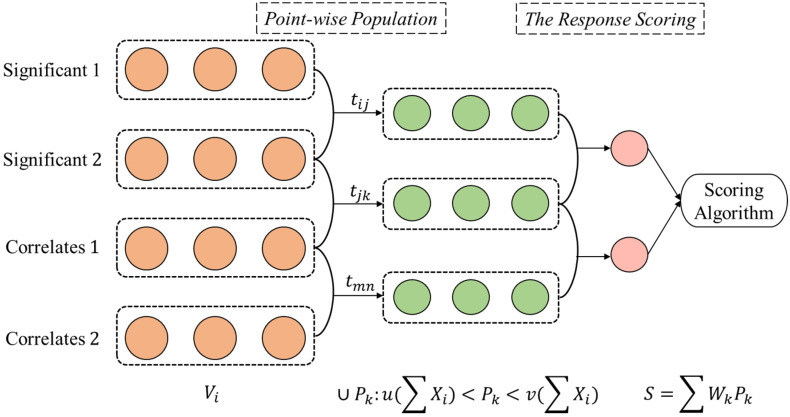
Process-quality machine learning framework overview.

The overall workflow for generating the quantitative process-quality neural network model is presented in Figure 5.

The point-wise population in Figure 5 generates scattered paired input parameters within the confidence interval. The generated data will also be grouped, thus ultimately combining with the response scoring algorithm to construct the deposition shape quantitatively. Therefore, the significant analysis of the input process parameters is required for proceeding with the later steps. Therefore, a stepwise regression model was initially conducted. This model takes a step-by-step iterative construction of regression models that involves all situations and possible variables that can be used in the final model. The simplified, reduced model for bead width contains the significant parameters: current (I), voltage (U), and speed. Hence, an ANOVA and F-test (at *α* = 0.05) were conducted to verify the importance of these variables and justify the individual importance level. From Table 2, current (I), voltage (U), speed, and their interacted terms are significant for bead width since the one-way factors display *p*-values smaller than 0.05.

In addition, a stepwise regression for analyzing the bead height was also conducted by adding or removing potential explanatory variables and testing for statistical significance iteratively. After the stepwise regression, the three process parameters present significantly varying bead height. The ANOVA test of these three variables is presented in Table 3.

These significant variables were obtained for constructing the neural network framework. The quantitative multi-variant process-quality model will use the obtained significant variables to describe the intercorrelated effect on the bead shape. The decomposition of the collected data into three-dimensional space (formatted in (I, U, S, BW, BH, DOP)) will be first introduced. The decomposition is as follows:

$$[I,\;U,\;S,\;\mathrm{BW}]\;\to :\;\{[I_{i},\;y_{I_{i}},{\;\mathrm{BW}}_{i}],\;[U_{i},\;y_{U_{i}},\;{\mathrm{BW}}_{i}],\;[S_{i},\;y_{S_{i}},\;{\mathrm{BW}}_{i}]\text{\}}=\{P_{I},P_{U},P_{S}\text{\}}$$

The algorithm below shows the construction of the machine learning framework from the decomposed point-wise input variables {$P_{I},P_{U},P_{S}$}.
**Algorithm**: Machine learning framework for decomposed vectorized data.**Algorithm: Machine Learning Framework Construction****Input**: Decomposed Variables {$X_{i,j}$} from {$X_{i}$}: $X_{i,j}\to {\mathbb{R}}^{3}$**Output**: Point-wise Populated ($U_{i}$), and Scored Response {S}1. Initialize ${\mathrm{Poly}}_{i}=\vec{X_{i,j}}$, ∀ $X_{i,j}\in${$X_{i}$} 2. $\mathrm{Populate}\;P_{k,i}$: $P_{k,i}\in \left[{\mathrm{Poly}}_{i}-B_{i},{\mathrm{Poly}}_{i}+B_{i}\right]$
3. $D_{k,i}=\mathrm{dist}(P_{k,i},{\mathrm{Poly}}_{i}$)4. $w_{k,i}\propto 1/D_{k,i}$
5. $S\left(k,i\right)\;:=\sum_{i}w_{k,i}P_{k,i}$
6. Weight function = $\arg min\|X_{i,j},S{\left(k,i\right)\|}^{2}$7. 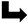 Update {$w_{k,i}$} and {$B_{i}$}8. Return $U_{i}$, S

In this algorithm, $B_{i}$ represents the bandwidth of the point-wised populations, and $w_{k,i}$ stands for the weight on these populations. This algorithm presents a systematic approach to develop the machine learning framework and create the quantitative response using the vectorized experimental data. The prediction of the shape (width, height, and DOP) can be described as follows:

***Objectives***: $min\;var\left(dist\left(\left(x_{i}+\Delta_{i},y_{i}\right),f\right)\right)$
***Randomized***: {$\Delta_{1}$, $\Delta_{2}$,…, $\Delta_{i}$}
$Populate\;:=\left(\left[x_{i},\;y_{i}\right]\right)$

$$dist_{i}=abs\left|\left(x_{i}+\Delta_{i},y_{i}\right)-f\left(\left[x_{i}+\Delta_{i},y_{i}\right]\right)\right|$$

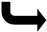
 **Solution**: Shape Response = $average\left(f\left(\left[x_{i}+\Delta_{i},y_{i}\right]\right)\right)$

{$x_{i}$} represents the vectorized process parameter inputs, {$y_{i}$} represents the {$x_{i}$} corresponding vectored axis value, and {$\Delta_{i}$} represents the deviation on the process parameters inputs.

The printable zone (collection of the optimal process parameters) is obtained from a multi-objective optimization machine learning process using the developed process-quality response model with a desired response level as the input. {$Z_{i}$} represents the corresponding response from vectorized process parameter - {$x_{i}$}. {$f_{k}$} represents the response function for $k^{th}$ item. The optimization process can be described as follows:

***Objectives***: $min\;var\left(diff\left(Z_{i},\bar{Z}\right)\right)$, $\bar{Z}$: targeted response
***Randomized:*** {$x_{i}$}
$Populate\;:=\left(\left[x_{i},\;y_{i}\right]\right)$

$$Z_{i}=f_{k}\left(\left[x_{i},\;y_{i}\right]\right)$$

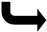
 **Solution**: Printable Zone {$x_{i}$} = $range(den\left\{x_{i}{|}_{f_{k}}\right\}>0.8)\cap^{\;}range(den\left\{x_{i}{|}_{f_{k+1}}\right\}>0.8)$.

## 3. Result and Discussion

**Figure 6:**
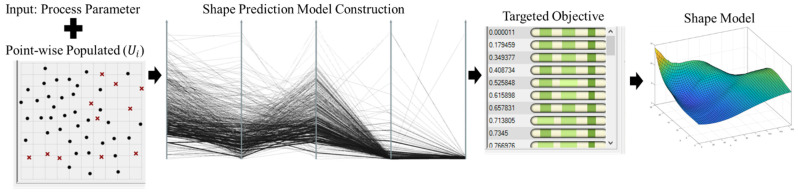
Deposition shape response construction through implemented machine learning framework.

The proposed machine learning framework has been implemented through Rhino Grasshopper. Figure 6 presents the working process through the implemented machine learning framework.

The deposition shape response model can be obtained through optimization iteration on the bandwidth of the point-wised populations and the weight on these populations through the proposed algorithm.

**Figure 7:**
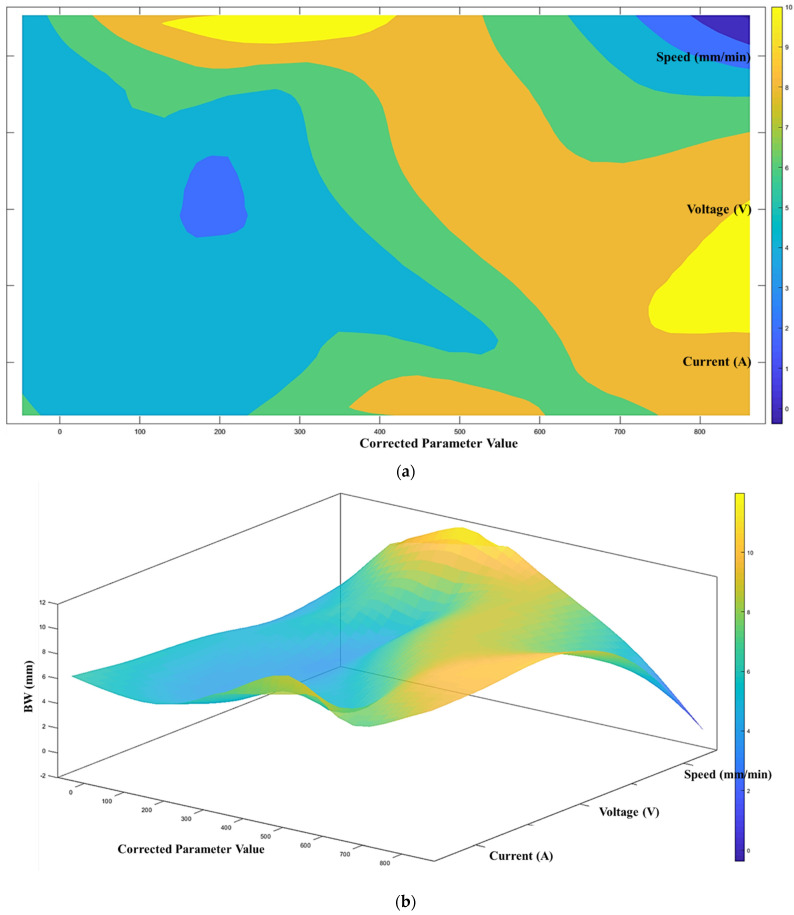
(**a**) Process-quality model (bead width) using current, voltage, and speed (contour), (**b**) process-quality model (bead width) using current, voltage, and speed (3D).

**Figure 8:**
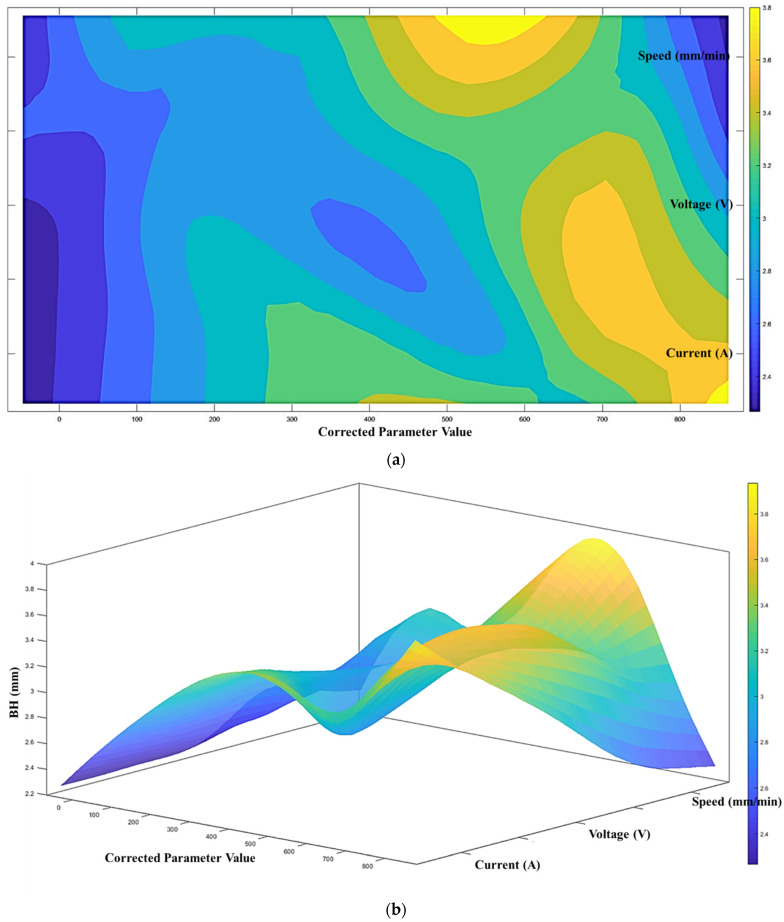
(**a**) Process-quality model (bead height) using current, voltage, and speed (contour), (**b**) process-quality model (bead height) using current, voltage, and speed (3D).

**Figure 9:**
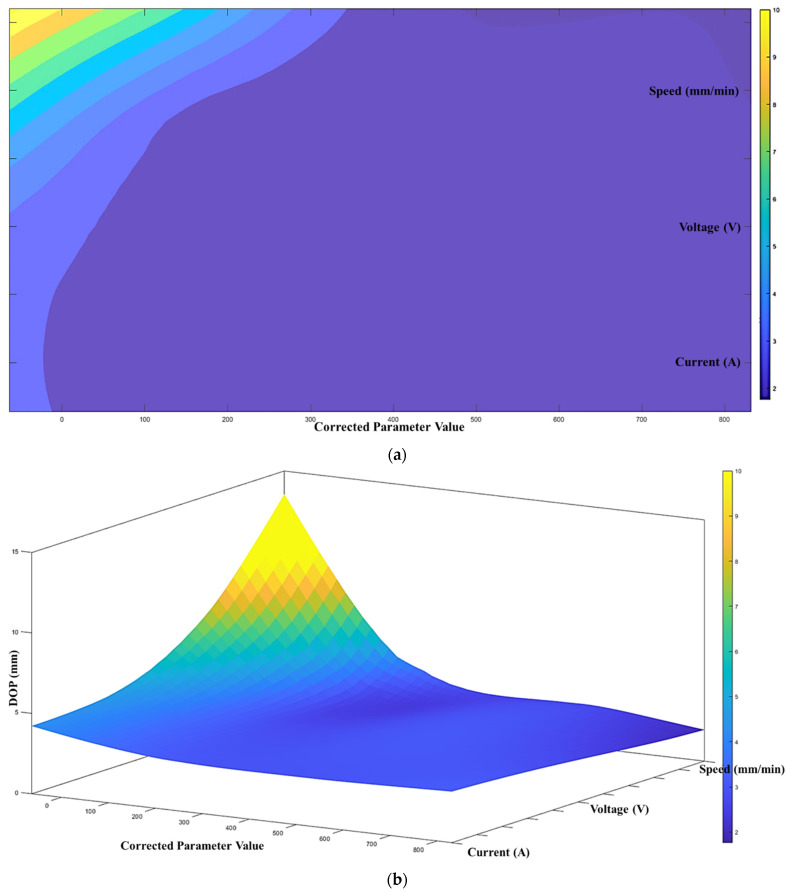
(**a**) Process-quality model (DOP) using current, voltage, and speed (contour), (**b**) process-quality model (DOP) using current, voltage, and speed (3D).

According to the proposed architecture of the quantitative process-quality model, the deposited bead response graph with regards to the three process parameters is presented in Figure 7, Figure 8 and Figure 9. Since all three process parameters are highly correlated and individually significant to the response, a simple regression model cannot describe such variable-response relation. Therefore, a quantitative response model is constructed based on the proposed machine learning framework to describe the complex model among the variables.

Figure 7, Figure 8 and Figure 9 show the proposed shape response model from the process variables based on the novel machine learning framework. The statistical measure ($R^{2}$) that represents the proportion of the variance for the developed response models (Figure 7, Figure 8 and Figure 9) are presented in Table 4. In addition, the $R^{2}$ from multi-variant linear regression models, and the traditional ANN classification methods are presented for comparison.

From Table 3, the proposed model shows the most accurate representation of the deposition bead shape compared with the multi-variant linear regression and the ANN classification models. This can indicate that the proposed machine learning framework performs better than the traditional machine learning methods. As mentioned in the previous section, the developed response model will be used to determine the printable zone to control the shape within a satisfactory level and predict the deposition bead shape.

**Figure 10:**
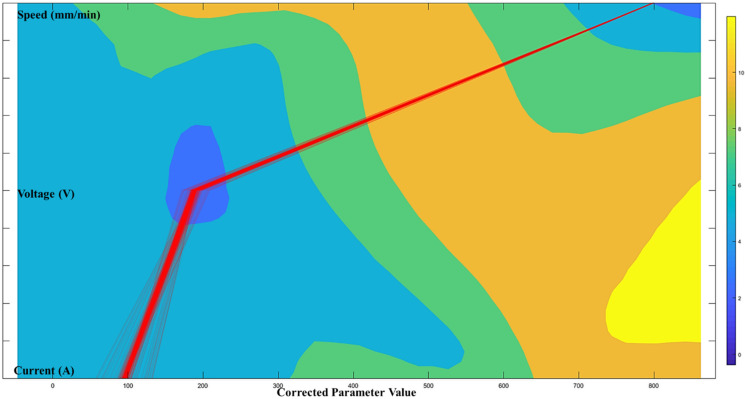
Optimal printable parameters for controlling BW = 3.7 mm in a 98% confidence interval.

**Figure 11:**
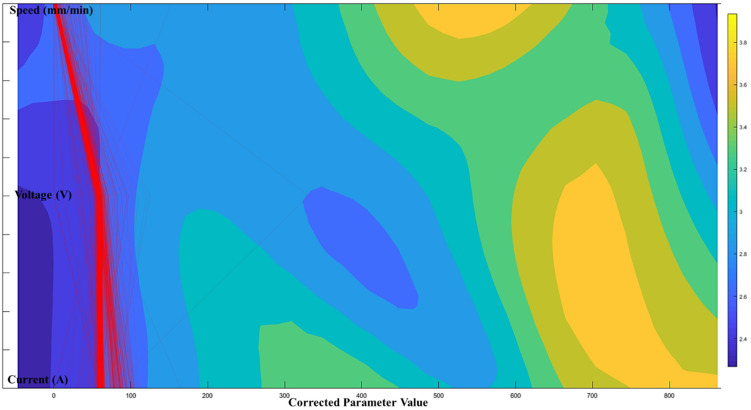
Optimal printable parameters for controlling BH = 2.6 mm in a 98% confidence interval.

**Figure 12:**
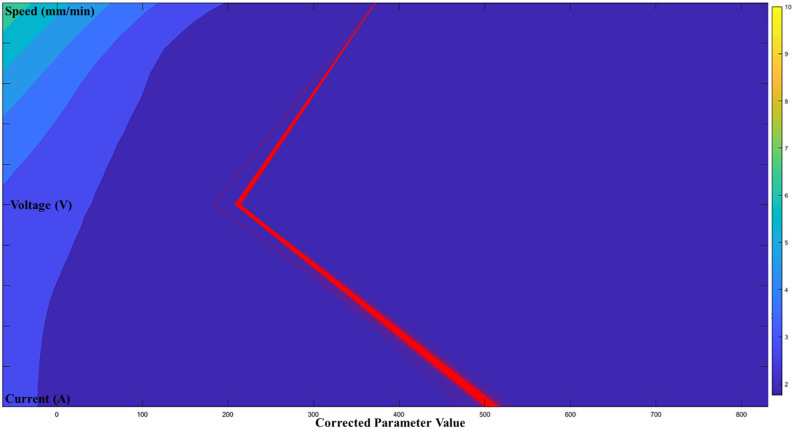
Optimal printable parameters for controlling DOP = 2.5 mm in a 98% confidence interval.

Figure 10, Figure 11 and Figure 12 show the optimal selection of the process parameters based on the desired level of bead shape from the developed multi-dimensional surface response using the multi-objective optimization method mentioned in the previous section. The red lines represent the optimal input dataset for achieving a desired geometry.

**Figure 13:**
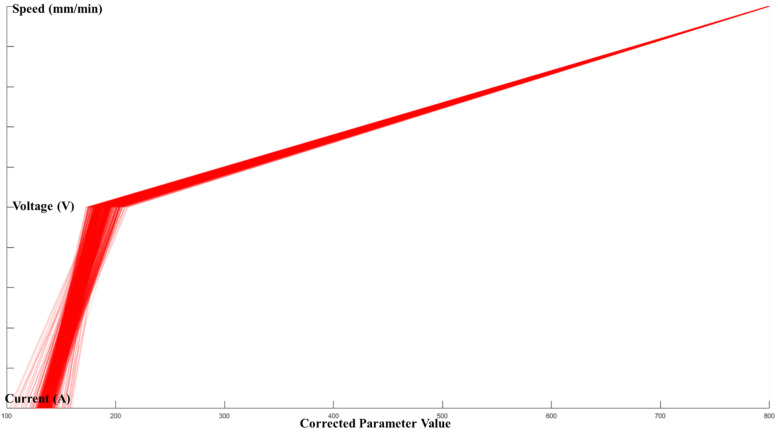
Optimal printable zone for controlling BW = 3.7 mm, BH = 2.6 mm, and DOP = 2.5 mm in a 95% confidence interval.

Figure 10, Figure 11 and Figure 12 present all process parameter combinations that derive the deposited shape to different desired condition: BW = 3.7 mm, BH = 2.6 mm, and DOP = 0.59 mm. The ultimate determination of the optimal process parameters to achieve the desired deposition shape when considering all conditions requires conducting a global search through three developed response models. The printable zone plot for the obtained optimal parameter dataset (current, voltage, and speed) is sketched in Figure 13. Based on the threshold of the density plot on each process parameter, the optimal range of the process parameters can be further refined.

**Figure 14:**
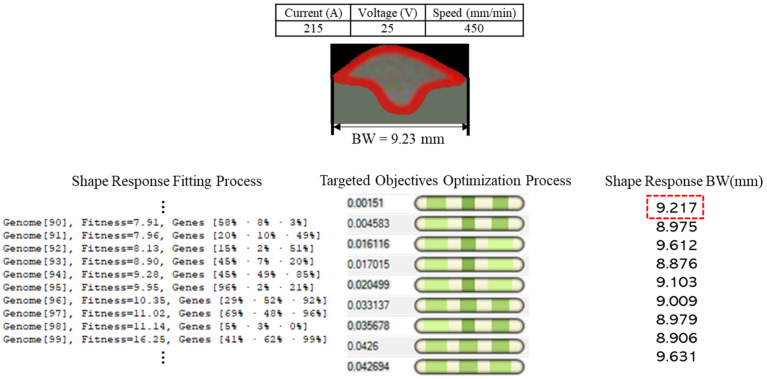
Bead width prediction process with experimental validation.

The above figure presents an optimal printable zone for controlling the deposited bead shape at a certain level with a 95% confidence interval. Note, the *x*-axis in Figure 13 represents the corrected parameter value. This optimal printable zone can also be verified by including the parameter setting as indicated by Subramanian [24]. To further validate the result, three weld bead profiles are measured using PlotDigitizer Software based on the different experimental set up are obtained and compared with the prediction bead shape. The shape prediction process and the measured bead width are illustrated through Figure 14.

Figure 14 provides a shape prediction process using the developed model and the defined process parameters. The targeted objective is minimized, and the ultimate result would provide the predicted bead deposition shape. As shown in Figure 14, the measurement data is also provided to validate the prediction. Table 5 below shows the prediction and measurement variation through different process parameters.

From Table 5, the maximum variation between the prediction and the measurement is less than 5% which can further validate the accuracy of the proposed process-quality model through the novel machine learning framework. Compared with the regression and the ANN models, the proposed machine learning model achieves the highest accuracy on the developed prediction models. The shape prediction model performance accuracy is 99.8%, 99.1%, and 99.6% for bead width, 99.6%, 98.8%, and 95.7% for bead height, 99.8%, 99.5%, and 97.5% for DoP based on the experimental data.

## 4. Conclusion and Future Recommendations

The developed machine learning framework would bring the following benefits compared with other existing analytical methods:

- Prediction models are eased to be adapted based on the increasing amount of collected data.
- Prediction provides a quantitative analysis of the process-quality relation.
- The reversely computed printable zone provides a numerical control to the WAAM system.

The proposed quantitative shape model from the various process parameters can provide the numerical prediction on the bead shape and can lead to the following conclusions:

- An increase in the current would result in a wider bead width and height.
- An increase in the voltage would result in a wider bead width, and the bead height would first increase then decrease.
- An increase in the speed would first result in a wider bead width and then reduce the width, and it shows similar changes for the bead height. In contrast, the depth of penetration would decrease with the increase in the speed.
- Wider bead width will lead to a smaller bead height and a larger depth of penetration.

The precise prediction of the deposited bead shape from the selection of the process parameters can be achieved through the proposed quantitative process model using the proposed machine learning framework. The multi-dimensional neural network model can rapidly react to data changes and systematically demonstrate the multi-dimensional connections among the process-quality network. In addition, the reverse computed optimal printable zone from the desired process quality level can also be deduced. This quantitative process method can comprehensively oversee such complex processes with multi-process variabilities.

The proposed quantitative predictive model of the deposition geometry can pave the path in full feedback controlling the multi-layering process. The lack-of-built volume and the over-built volumes can be compensated by correctly adjusting the processing parameters in the real-time printing process through the developed model. In addition, the proposed machine learning framework is eased to adopt to other material processing systems.

## Figures and Tables

**Table 1:** Existing research on process parameters effect on build qualities.

Analytical Process Parameters	Research Output
Wire feed speed (WFS), travel speed (TS), the ratio between WFS and TS [9]	The ratio between WFS and TS is the most significant process parameter for controlling heat input Increased heat input will cause added material flow to distort the width, height, and weld penetration area of the beads
Heat input [11]	Increased heat input will result in coarse grain, homogeneous microstructure but lower material hardness
Deposition direction, nozzle tip distance, and gas pressure [12]	Pressure and current, nozzle tip distance has the most significant effect on tensile strength Current has the most significant effect on hardness
Path planning trajectory [13]	WFS, welding current, cooling time, and interlay temperature affects dimensional accuracy and surface finish
Vibration [14]	Increased vibration acceleration will decrease the average grain size as well as homogenize the grain distribution Vibration can reduce porosity and increase tensile strength
Air cooling, idle time [15,16]	Air jet is not effective at preventing an increase in the substrate temperature Air jets can effectively reduce the overall increase in temperature

**Table 2:** ANOVA analysis of parameter’s effect on bead width.

Term	Coef	SE Coef	T-Value	*p*-Value	VIF
**Constant**	4.050	0.488	8.30	0.000	
**I (Amps)**	0.00840	0.00274	3.06	0.003	1.28
**U (Volts)**	0.03083	0.00475	6.49	0.000	1.21
**Speed (mm/min)**	−0.0780	0.00409	−1.91	0.042	1.41

**Table 3:** ANOVA analysis of parameter’s effect on bead height.

Term	Coef	SE Coef	T-Value	*p*-Value	VIF
**Constant**	3.566	0.172	20.78	0.000	
**I (A)**	−0.002465	0.000965	−2.55	0.014	1.28
**U (V)**	0.00506	0.00167	3.03	0.004	1.21
**Speed (mm/min)**	−0.00654	0.00144	−4.55	0.000	1.41

**Table 4:** Statistical measures of the proposed model, regression, and ANN classification method.

	Bead Width Model	Bead Height Model	Depth of Penetration Model
**Proposed Model**	0.997	0.993	0.9853
**Regression**	0.9237	0.7181	0.8643
**Tradition ANN**	0.464	0.857	0.80

**Table 5:** Shape variation between prediction and measurements.

Sample No.	Current (A)	Voltage (V)	Speed (mm/min)	BW (mm)	BH (mm)	DOP (mm)
1 Measured	215	25	450	9.23	3.12	5.71
1 Predicted				9.217	3.108	5.721
2 Measured	215	27	450	9.3	3.41	2.54
2 Predicted				9.218	3.451	2.551
3 Measured	250	26	600	8.56	3.3	2.45
3 Predicted				8.528	3.158	2.51
